# Climatically promoted taxonomic homogenization of macroinvertebrates in unaffected streams varies along the river continuum

**DOI:** 10.1038/s41598-023-32806-y

**Published:** 2023-04-18

**Authors:** Marie Zhai, Jindřiška Bojková, Denisa Němejcová, Marek Polášek, Vít Syrovátka, Michal Horsák

**Affiliations:** 1 Department of Botany and Zoology, Masaryk University, Kotlářská 2, 611 37 Brno, Czech Republic; 2 T. G. Masaryk Water Research Institute, p.r.i., Podbabská 2582/30, 160 00 Prague 6, Czech Republic

**Keywords:** Community ecology, Ecology, Ecology, Limnology

## Abstract

Biotic homogenization appears to be a global consequence of anthropogenic change. However, the underlying environmental factors contributing to homogenization are difficult to identify because their effects usually interact and confound each other. This can be the reason why there is very little evidence on the role of climate warming in homogenization. By analysing macroinvertebrate assemblages in 65 streams that were as close to natural conditions as possible, we avoided the confounding effects of common anthropogenic stressors. This approach resulted in revealing a significant effect of increased temperature (both summer and winter) on changes in macroinvertebrate compositional over the past two decades. However, homogenization was significant only at opposite ends of the river continuum (submontane brooks, low-altitude rivers). Surprisingly, species of native origin predominated overall, increasing in frequency and abundance (“winners”), while only a minority of species declined or disappeared (“losers”). We hypothesise that undisturbed conditions mitigate species declines and thus homogenization, and that the temperature increase has so far been beneficial to most native species. Although we may have only captured a transitional state due to extinction debt, this underscores the importance of maintaining ecological conditions in stream to prevent species loss due to climate change.

## Introduction

Although biotic homogenizations very probably occurred locally during the whole evolutionary history^1^, anthropogenic activities in the industrial era seem to make it a global scale phenomenon^2^. In such temporal changes of biotic communities, common taxa spreading to new sites (“winners”) often replace spatially restricted taxa (“losers”), which increases a taxonomic and/or functional similarity among communities over time^3^. Although the exact mechanism behind homogenization is not universal^4^, environmental changes play a key role in the whole process. It may either facilitate establishment of a “winner” in a community^5,6^, or directly filter “winners” and “losers” from species pools. The “winners” are typically non-native species^3,7^, but they can be also native species^8,9^. In freshwaters, various stressors have been recognized to cause biotic homogenization, i.e., damming^7,10^, increased productivity^11^, salinization^12^, and changes in land use^13^. The communities with more similar compositions and functions potentially display decreased stability and resilience to further environmental changes^14,15^.

Temperature is one of the key environmental variables, which affects all levels of organization in biota from cells to communities^16,17^. The global increase in temperature^18^, impacting also running water systems^19^, can cause a decline in cold-adapted taxa and an increase in taxa adapted to higher temperatures^20,21^. Thus, it potentially leads to major compositional changes and functional restructuring of local communities^22,23^. However, whether and to what extent such climatically induced changes contribute to biotic homogenization has been only poorly studied^24,25^. That is mainly because numerous anthropogenic changes, such as land use, pollution and others, may largely confound the effect of increasing temperature^26–28^.

In this study, we analyse data on benthic macroinvertebrate assemblages repeatedly collected at 65 streams along the river continuum in the three time periods (1997–2000, 2007–2008, and 2015). Two main characteristics of this dataset make it especially suitable for exploring biotic homogenization in relation to the increase of temperature. First, we included only sites in an as-close-to natural state as possible to minimize the effect of common anthropogenic stressors. Second, our network of sampling sites evenly covers a large part of the river continuum from submontane brooks to lowland rivers. In contrast, most previous reports on long-term compositional changes in running waters have focussed only on a restricted section of the river continuum^23,29^. As different sections of the river network vary in proportion of cold-stenotherms, responses of macroinvertebrate assemblages to long-term changes are likely to differ^22,23^ or can lead to different degree of biotic homogenization^8^ along the river continuum.

Our primary goal was to test for the existence of compositional shifts with time leading to taxonomic homogenization (H1). As the main environmental conditions, selecting “winners” and “losers”, change in the longitudinal profile of streams, we expect that the degree of homogenization would differ along the river continuum (H2). Second, based on the evidence for both species profiting and losing due to the environmental changes in streams during the last decades, we assume homogenization to be caused equally by a decline of “losers” and an increase of “winners” (H3). And most importantly, as by including only close-to-natural sites, we hypothesise that temperature should appear as the main driving factor of the compositional changes through time (H4).

## Materials and methods

### Study area

**Figure 1:**
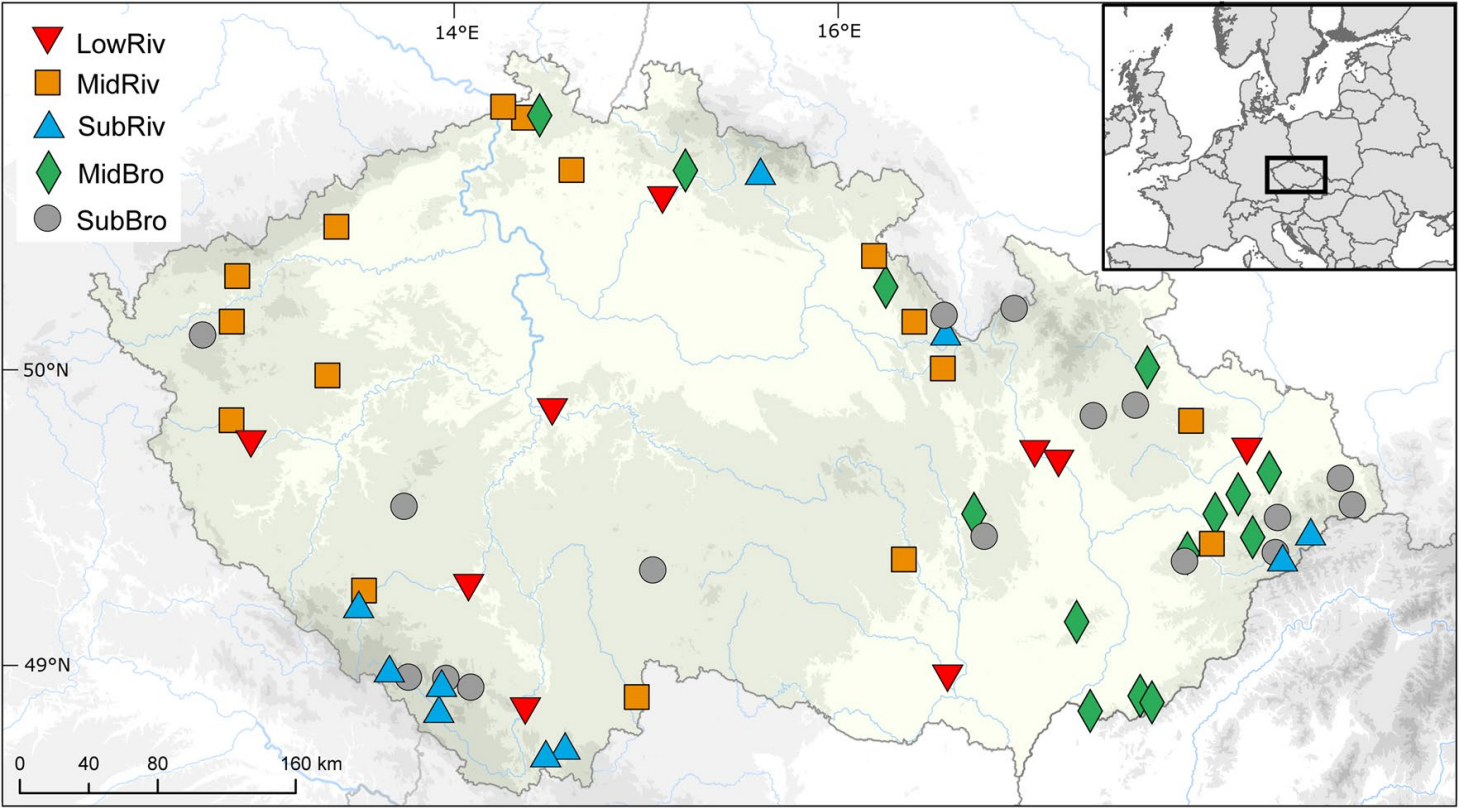
Locations of the sampling sites in the Czech Republic: low altitude rivers (LowRiv), mid-altitude rivers (MidRiv), submontane rivers (SubRiv), mid-altitude brooks (MidBro), and submontane brooks (SubBro). The map was created using Arcgis 8.3 program^59^.

We selected 65 streams of 2–7th stream Strahler order, distributed at altitudes of 150–800 m a.s.l. in all main river basins of the Czech Republic (Fig. 1). To avoid confounding effects of other anthropogenic stressors, the main criterion for selection was minimal anthropogenic influence in terms of physical conditions, including natural thermal and discharge regime, water quality and the characters of riparian zone and floodplain, all being as-close-to natural state as possible. Owing to their natural character all these sites were used for establishment of type-specific reference conditions according to requirement of the Water Framework Directive, 2000/60/EC^30^.

The study sites were classified according to their size (stream order according to Strahler, distance from source), catchment area and altitude into five stream types: submontane brooks (SubBro), submontane rivers (SubRiv), mid-altitude brooks (MidBro), mid-altitude rivers (MidRiv), and low altitude rivers (LowRiv) (Table 1). Unfortunately, there are no reference stream sites representing (a) lowland brooks and large rivers (such as lowland stretches of the Morava, Elbe and Vltava Rivers), which suffered morphological degradation and poor water quality, and (b) high mountain brooks and rivers, where we lack information on the past impact of acidification^30^.

**Table 1:** Basic characteristics of the 65 stream sites classified into five types along a river continuum: low altitude rivers (LowRiv), mid-altitude rivers (MidRiv), submontane rivers (SubRiv), mid-altitude brooks (MidBro), and submontane brooks (SubBro).

Stream type	N	Altitude (m a.s.l.)			Distance from source (km)			Catchment area (km^2^)			Strahler stream order		
		Min	Med	Max	Min	Med	Max	Min	Med	Max	Min	Med	Max
LowRiv	9	175	236	461	51	94	212	816	1862	4301	6	7	7
MidRiv	16	157	370	472	16	39	81	47	199	659	4	5	6
SubRiv	10	485	569	895	10	19	53	55	100	479	5	5	6
MidBro	14	257	390	470	2	3	14	1	4	38	2	3	4
SubBro	16	488	601	801	1	4	9	2	5	33	2	3	4

### Biotic data

Sampling was done during spring (April–May) and autumn (September–October) in three periods: 1997–2000 (period 1), 2007–2008 (period 2) and 2015 (period 3), using the same standardized methodology^31^. Benthic macroinvertebrates were sampled using a multi-habitat sampling method, i.e., taking semi-quantitative 3-min kick samples gathered with a hand net of 500 μm mesh size^32^. The sampling was done proportionally in all mesohabitats (e.g. riffle, pool, macrophytes, woody debris) within a typical stream stretch of up to 100 m long. This sampling method showed a high precision when compared to other methods^33^. The samples were preserved in 4% formaldehyde and sorted under stereomicroscope in the laboratory.

Macroinvertebrates were identified mainly to species, except of Diptera, some Oligochaeta and Coleoptera that were identified to genera. Because of variations in some aspects of taxonomic resolution through time, some species were pooled, using operational taxonomic units. This concerned some species groups of Ephemeroptera, Plecoptera, Trichoptera and Oligochaeta, with unclear reliability of identification in the 1990s. Chironomidae were excluded from the dataset due to varying taxonomic resolution between the periods.

### Environmental variables

Water quality parameters were measured six times per year during the respective years of macroinvertebrate sampling. They included water temperature, dissolved oxygen and oxygen saturation in water, biological oxygen demand (BOD_5_), pH, acid neutralising capacity (ANC), electrical conductivity and nutrient concentrations (TP, PO_4_-P, NO_3_-N, NH_4_-N) as required for ecological status assessment according to the Water Framework Directive, 2000/60/EC^34^. The mean roughness of bed substrate (Phi) was estimated by the cover of individual particle sizes^35^. Share of riffles (Riff) was assessed based on visual estimations in the sampling stretches during the macroinvertebrate sampling.

Three climatic variables, the average air temperatures in January and July (T_Jan, T_Jul) and annual precipitation (Prec), were calculated based on values of daily average temperature and daily precipitation in the year of sampling and two consecutive years prior to sampling, using gridded data provided by the Czech Hydrometeorological Institute^36^.

Four variables describing land use were chosen: areas of unfavourable surfaces (Un_surf), artificial surface (Ar_surf), arable land (Ar_land) and forested area (Fore). They were computed based on the information from the CORINE Land Cover system (for details see Supplements to Methods).

### Statistical analyses

Prior to statistical analyses, the biotic data from spring and autumn within a sampling period were combined for each sampling site. To remove the influence of very rare species in all analyses, we included only the species recorded at least in three samples.

#### Compositional changes

To visualize the compositional change in the macroinvertebrate assemblages with time, we performed Non-Metric Multidimensional Scaling (NMDS) of all samples, using Bray–Curtis dissimilarities of ln(x + 1) transformed abundance data, and drew trajectories of the assemblage developments in the ordination space as arrows.

As a measure of beta-diversity, we used the mean distance of sites to stream type centroids, calculated as Bray–Curtis dissimilarities of ln(x + 1) transformed species abundance data. We tested the homogeneity of multivariate dispersions among the three sampling periods using one-way ANOVA, both for the whole set of sites and also separately for each stream type.

We identified the species most responsible for the compositional changes between the time periods (1–2, 2–3, and 1–3), by categorizing the relative changes in species abundance and frequency. The relative change in species abundance between two periods was calculated using ln(x + 1) transformed abundance data (for details see Supplements to Methods) and it ranged from 1 (for the species that newly occurred at sites) to -1 (for the species that completely disappeared from all sites). For example, species with the relative change in abundance of 0.25 had 25% lower mean abundance in the former than in the latter period. We categorized species according to their relative change in species abundance between the periods 1–3, considering those within the intervals of [(-1)–(-0.5)] as “losers”, and those within the intervals of [0.5–1] as “winners”. Please note that our definition of “winners” and “losers” is arbitrary and can be used only for mutual comparisons in this study. To assess the differences in species composition along the river continuum we analysed the relative change in species abundance for each of the stream types separately.

#### Effect of environmental variables

To test the effects of environmental variables, we performed distance-based Redundancy Analysis (db-RDA), using Bray–Curtis dissimilarities of ln(x + 1) transformed abundance data and forward selection procedure for environmental variables. The significance of each potentially added variable was tested by 999 permutations restricted within sites, as we were interested in the temporal within-site variability. Variables with the highest contribution to the explained variation in the species data were added sequentially in the model if the p-value of their contribution was < 0.05. We also partitioned the pure and shared effects of temperature (T_Jan, T_Jul) and other significant environmental variables in the models. Db-RDAs were done both for the whole set of sites and separately for each stream type.

All analyses and visualizations were performed in R version 3.4.4^37^, using packages vegan 2.5–6^38^ and ggplot2^39^ packages.

## Results

**Figure 2:**
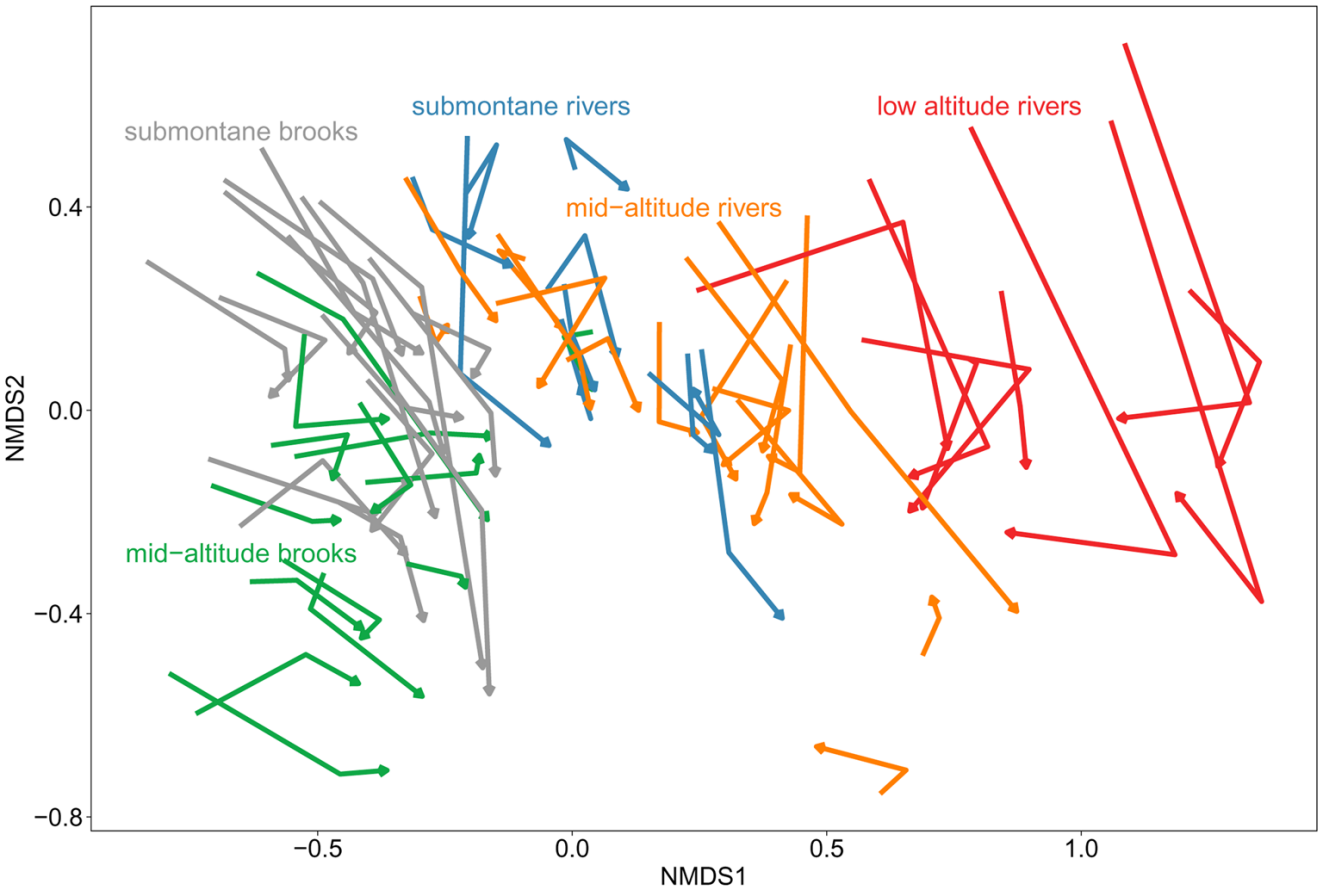
Compositional shift of macroinvertebrate assemblages in the 65 stream sites repeatedly sampled in 1997–2000 (period 1), 2007–2008 (period 2) and 2015 (period 3). Species data were analysed by Non-Metric Multidimensional Scaling (NMDS) on Bray–Curtis dissimilarities of ln(x + 1) transformed abundances; stress = 0.153. The sites are classified into five types along a river continuum. Samples from the sampling periods are connected by an arrow for each site, heading towards the youngest time period.

**Figure 3:**
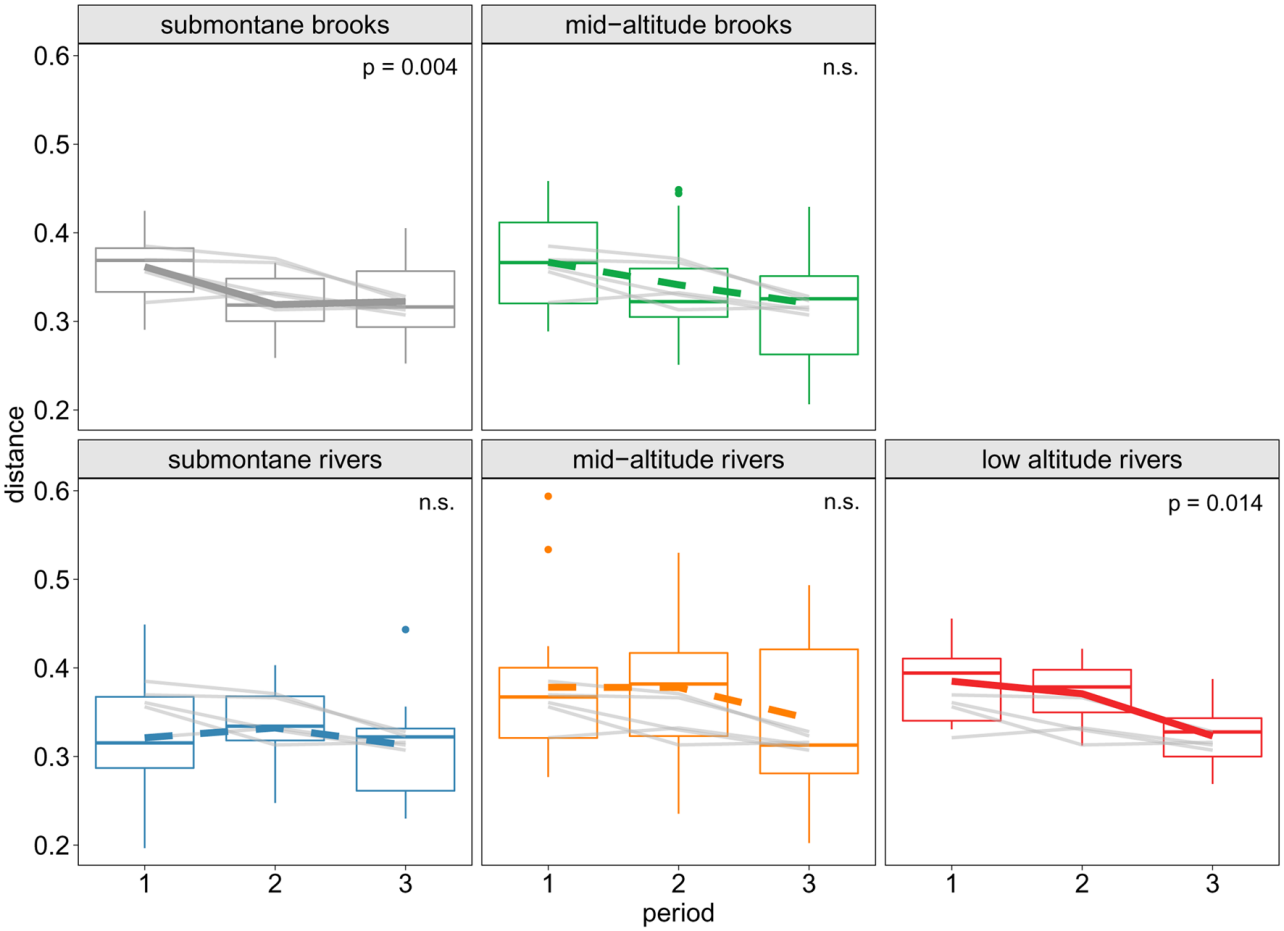
Beta-diversity of macroinvertebrate assemblages in the 65 repeatedly sampled stream sites in 1997–2000 (period 1), 2007–2008 (period 2) and 2015 (period 3). Streams were divided into five types along a river continuum to be analysed separately. Beta-diversity of each group was counted as mean distance of sites to the group centroid created by principle coordinate analysis (PCoA), using Bray–Curtis dissimilarities of ln(x + 1) transformed abundance data. Homogeneity of multivariate dispersions in the three periods was tested using one-way ANOVA. n.s.—*p* > 0.05.

The main gradient in taxonomic composition was associated with the stream typology based on stream size and altitude (Fig. 2), reflecting the river continuum. Individual sites showed considerable shifts in species composition over the time, heading closer to each other in the NMDS plot (Fig. 2), which might indicate an overall increase in assemblage similarity among sites. Despites this, total beta-diversity did not significantly differ between the three periods (*p* = 0.1742), suggesting only a trend to taxonomic homogenization among all the study sites. However, when beta-diversity was analysed for stream types separately, it significantly differed in submontane brooks (*p* = 0.0042) and lowland rivers (*p* = 0.0139) (Fig. 3).

Comparing the species composition between the first (1997–2000) and the third (2015) period, there was a relatively large number of species that newly occurred or increased their mean abundance at sites by up to 50%, and we considered them as “winners” (Table 2). In total, there were 158 macroinvertebrate species classified as “winners”, most of them (ca 79%), however, only in one or two stream types (Supplementary Table S1). Only seven taxa showed as “winners” in all stream types. There were only three non-native species among “winners”: planarian *Girardia tigrina*, gastropod *Potamopyrgus antipodarum*, and isopod *Proasellus coxalis*, all of them increased only in a single stream type. On the other hand, only several species (from one to five species) disappeared from the sites in each stream type, all of them being rare or relatively rare (frequency of 3–5 in the first period). Abundance of some other species (from four to nine species), decreased by more than 50% in a stream type. These two groups of diminishing species were considered “losers”, summing up to 37 spp. in total (Supplementary Table S2). The species frequencies seemed to change less than species abundances among the sampling periods but they showed similar patterns (Supplementary Fig. S1).

**Table 2:** Number of species recorded in each of five stream types and classified based on their relative change in their mean abundance between 1997–2000 (period 1) and 2015 (period 3) into intervals. Species appearing in the intervals [1–0.5] were arbitrary considered “winners” and “losers”. For calculations of the relative change and detailed species distributions see Supplements to Methods and Supplementary Fig. S1.

Stream type	Total N species	Intervals of relative change in species abundance between 1st and 3rd time period									
		[1.00]	(1.00–0.75]	(0.75–0.50]	(0.50–0.25]	(0.25–0.00]	− (0.00–0.25]	− (0.25–0.50]	− (0.50–0.75]	− (0.75–1.00)	− [1.00]
LowRiv	127	23	16	23	23	14	11	7	2	5	3
MidRiv	162	29	23	41	23	20	11	8	3	1	3
MidBro	125	20	15	30	22	20	7	5	4	1	1
SubRiv	124	11	11	20	15	25	22	10	8	0	2
SubBro	124	22	13	20	27	16	11	4	6	2	3
		"winners"							"losers"		

**Figure 4:**
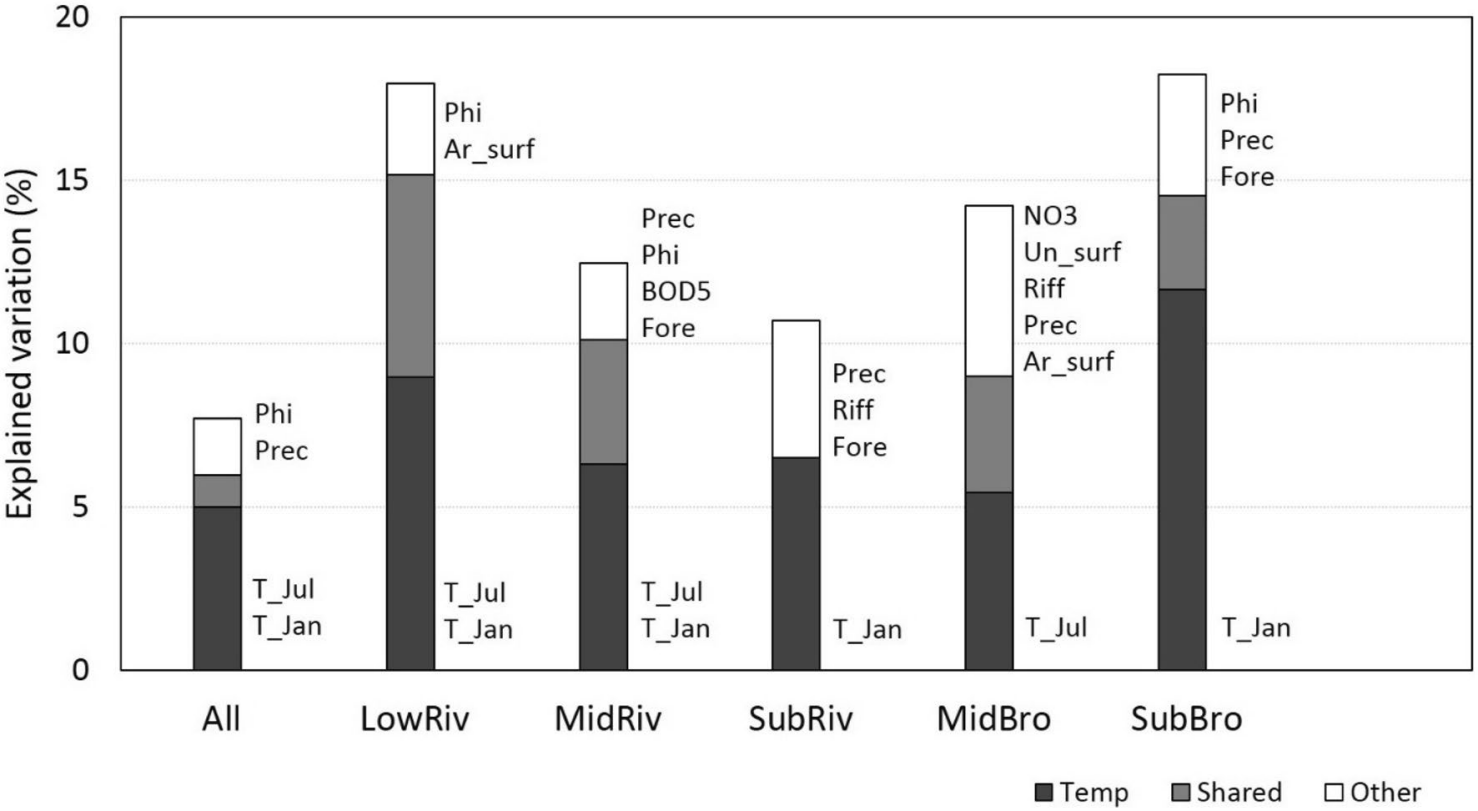
Total, pure and shared variation explained by temperature and other environmental variables in macroinvertebrate assemblages of the 65 stream sites compared between 1997–2000 (period 1) and 2015 (period 3). Counted as R^2^_adj._ by Distance-Based Redundancy Analysis (db-RDA) using Bray–Curtis dissimilarities of ln(x + 1) transformed abundance data, for the total dataset and separately for five stream types: low altitude rivers (LowRiv), mid-altitude rivers (MidRiv), submontane rivers (SubRiv), mid-altitude brooks (MidBro), and submontane brooks (SubBro). Significant environmental variables: mean air temperatures in January and July (T_Jan, T_Jul), annual precipitation (Prec), roughness of bed substrate (Phi), concentration of nitrates (NO_3_), artificial surfaces (Ar_surf), unfavourable surfaces (Un_surf), share of riffles (Riff), forested area (Fore), biochemical oxygen demand (BOD5). The models were significant at *p* < 0.01, permutations were restricted to sites. Temp—pure effects of temperature variables; Shared—effects shared by temperature and other environmental variables; Other—pure effects of other environmental variables.

Environmental variables explained 7.1% of the total variance for the whole dataset and 10.7–18.3% of the total variance in individual stream types (Fig. 4). Temperature variables (either July or January air temperatures, or both) were significant in the whole dataset (pure effect of 5.0%) and all stream types (pure effects of 5.5–11.6%). Pure effects of other environmental variables were lower than those of temperature: 2.7% in the whole dataset and 2.3–5.2% in individual stream types. Roughness of bed substrate and annual precipitation sums were the most significant of the other environmental variables. The effects shared between temperature and other significant variables ranged from 0 to 6.2% in individual stream types. The changes in the significant environmental variables at individual sites among the three sampling periods are given in Supplementary Fig. S2.

## Discussion

### Homogenization along the river continuum

We found that composition of macroinvertebrate assemblages in the Czech reference stream sites has undergone a substantial change over the last two decades. This agrees with other studies reporting compositional changes in stream communities from different parts of the world^23,25,40^. Despite the change, we found no significant taxonomic homogenization on the whole river continuum scale, disproving our H1. However, beta-diversity among the studied periods showed a decreasing tendency with time, implying that the process that may in future eventually lead to homogenization on this regional scale has probably already started. We suggest that the close-to-natural conditions at our sites and naturally high between-site variation decelerate homogenization. Since multiple stressors in running water systems have typically synergic effects^16,25^, we might expect more significant homogenization to be associated with non-reference state. For example, macroinvertebrates in streams from a wide range of altitudes, but affected by common anthropogenic changes, experienced taxonomic homogenization^25^.

When the stream types were analysed separately, homogenization was significant at both ends of the river continuum (submonate brooks, lowland rivers) but not in the middle. This confirms our H2 that the degree of homogenization differs along the river continuum. It also suggests different speed of homogenization along the river continuum, which seemed relatively slower in the middle of the river continuum. This might correspond to the rare case when no signs of taxonomic homogenization have been found in mid-altitude streams in Wales, UK, over the period of 30 years^41^. In contrast, both submontane brooks and lowland rivers homogenized relatively fast (within a single decade given the grain of our study), although in the former it happened a decade earlier than in the latter. We could thus confirm the recognition that biotic homogenization is not a linear process^42^.

### The lack of “losers” and prevalence of “winners”

Most of the species clearly increased their abundance and frequency at our sites over the studied period, while the loss of species played only a negligible role. This partly contradicted our expectation (H3) because although species loss/decrease and species gain/decrease are not necessarily reciprocal, homogenization of natural communities usually involves both these processes^25,42^.

In direct association with climate change, a decline of cold-adapted species has been observed in freshwater assemblages^23,43^. We could not confirm a significant decline in cold-stenothermal species, although the proportion of cold-stenotherms was higher among the “losers” (ca 46%) than among the whole species pool (22.8%). The decline of cold-stenotherms (mostly plecopterans and trichopterans) accounted only for a very minor compositional change even in submontane brooks and rivers, where stenothermic species naturally dominate. However, as the mountain streams are most vulnerable to climate change, we might expect some more cold-stenothermic “losers” in streams at higher elevations (> 900 m a.s.l.^23,44^).

“Winners” constituted the dominant proportion of species in each stream type, suggesting an immigration credit^42^ and increasingly favourable conditions for many macroinvertebrate species. This was quite surprising because, although “winners” have been found to prevail in some freshwater assemblages^45,46^, in general, homogenization due to prevailing species gain has been only rarely reported^8,9^. Most importantly, the vast majority of the “winners” in our study (mostly trichopterans, dipterans, clitellates, but many others) were from regional species pools, while only a very minor fraction (< 2%) was of non-native origin, their increase being limited for only one stream type. That does not accord with the predictions of establishment of non-native macroinvertebrates in stream assemblages due to climate change^44^ and the typical cases of homogenization due to an increase of alien species, which induce extirpations of native species^4,7^.

Several studies have predicted or already observed an increase of warm-stenotherms and eurytherms in stream macroinvertebrate assemblages^20,23,46^. However, the increase of species in our study could not be explained by known species thermal tolerances^47^. It is a reminder that thermal preferences, being mostly derived from the known species distributions, are probably only a very rough estimates of species temperature niches. However, analysis of a possible role of other species traits (such as substrate preference, flow velocity, and sensitivity to trophic status^47^) for the success of “winners” is far outside the scope of this paper.

An increase of native species in assemblages may be a sign of recovery from disturbances^48^, as found in some European streams following the improvement of water quality since the end of 1980s^49,50^. At our sites with persistently close-to-natural conditions, an increase of some species might partly reflect a recovery of ecological state in surrounding streams. The increased number of suitable sites in the landscape might support the populations of native species by higher rates of dispersal.

### The role of temperature for homogenization

In our study, temperature appeared to be the most significant environmental factor explaining the between period compositional changes at sites. The significant temperature effects were found across the whole river continuum and in all individual stream types, confirming our H4. With only few exceptions^45,51^, the previous studies could not unequivocally assign long-term compositional changes in macroinvertebrate assemblages to climate change due to the confounding effects of changes in water quality, land use, or strong hydrological events^25,49,50^. As in our study, temperature was the environmental variable with the largest and most consistent temporal change, we could support the significant effect of temperature on the macroinvertebrate assemblages by a strong evidence.

Temperature affects organisms directly, modulating their metabolism and phenology^16,17^, and/or indirectly by changing the productivity of the whole system^45^. By either way, the increase in temperature allowed some previously temperature limited species to establish more abundant populations. The increase in winter temperatures, suggesting a relaxed limitation by cold extremes^52^, was most significant in submontane brooks and rivers, but performed across whole river continuum. The increase in warm summer temperatures was significant only in the middle and lower parts of the river continuum, having surprisingly positive effect on many species.

A long term systematic enrichment in stream macroinvertebrates has been explained by a higher primary production, leading to a higher complexity in food webs^45^. There are no data of primary production available for our sites, however, all the indicators of tropic status (total phosphorus, nitrates, BOD5) did not show any consistent trend that could account for the large increase in macroinvertebrates (Supplementary Table S3).

### Combined effect of increased temperature and changes in hydrology

The impact of climate change on running waters include the combined effects of temperature and flow alternations^53,54^. Unfortunately, direct measurements of discharge were not available for our sites. However, as precipitation (Prec) decreased with time at most of our sites, we assume a decreasing trend in discharge over the studied period. Structure of sediment (Phi) was another variable indicative of change in discharge. As both Prec and Phi were the most significant explanatory variables after temperature, we suggest that the significant change in macroinvertebrate assemblages is partly also accountable to the change in discharge. In lowland rivers, the reduction in discharge favoured macroinvertebrates associated with finer sediments and those preferring slow flowing conditions, similarly as in some other European rivers^50^.

In small streams of mid-altitudes, the changes in discharge might have led to an occasional temporal drying during the last studied time period. Due to the humid continental climate, Czech streams are historically mostly perennial, however, there has been an increase in flow intermittence during the last few decades, most recently at an accelerating rate^55,56^. Drying is a strong environmental filter for aquatic fauna favouring only resistant and resilient taxa^57^ and promoting nested communities with higher dominance of ubiquitous taxa^58^, which might result in homogenization. However, there is an evidence that newly occurring intermittent conditions support a higher replacement of taxa in streams and thus increasing beta-diversity^56^. Occasional drying might hamper the increase of abundance and frequency in “winners”, decelerating the homogenization in mid-altitudes.

## Conclusions

To sum up, the increase of air temperature in our study led to a large compositional change and/or, depending on the position in the river continuum, also taxonomic homogenization, all of that mainly due to a large increase in abundance and gain of species. Macroinvertebrate assemblages have been clearly undergoing massive reorganization during the last 20 years but not yet decrease and loss of species. However, we might have, in fact, observed only a temporal transitional state of the increase due to an extinction delay^42^. Such transitional state may be followed by a decline in abundances and loss of species with further increase of temperature after reaching a threshold^51^. Massive decline is probable especially if the increase of temperature is combined with strong changes in discharge conditions^53,54^. Nevertheless, the future development in stream assemblages remains highly unpredictable. In accordance with Van Looy et al.^45^, our study suggests that loss of species may be largely prevented even despite the ongoing climate change by maintaining or improving the ecological quality of streams.

## Supplementary Information

Supplementary Information.

## Data Availability

Data are available from the authors upon reasonable request—contact Jindřiška Bojková (bojkova@sci.muni.cz).
