# Supplementary material for: Climatically promoted taxonomic homogenization of macroinvertebrates in unaffected streams varies along the river continuum
Source: Sci Rep. 2023 Apr 18;13:6292. doi: 10.1038/s41598-023-32806-y (PMC10113374; doi:10.1038/s41598-023-32806-y)

Supplementary Information

Supplements to Methods

- Land use

The layers best corresponding to the sampling periods were selected: CLC_2000 layer from 2000 for the period 1, CLC_2006 layer from 2006 for the period 2, and CLC_2012 layer from 2012 for the period 3. Individual surface types were expressed as area of each type in the riverside strip 250 m wide (on both banks) along the stream network to the maximum distance of 15 river kilometres upstream above the site, defined by the river basin perimeter.

- Calculation of relative change in abundance and frequency

First, for each species and each site, we calculated the number of individuals that remained at a site (a), the number of individuals that disappeared from a site (b), and the number of individuals that appeared at a site (c). Consider that the abundance of a species in the former period was 2 and in the latter it was 5. Then, 2 individuals remained at the site, 0 disappeared and 3 appeared there. Then we calculated for each species (OUT) the relative change in abundance as (A - B) / max (A + B, A + C), where "A", "B" and "C" represent the means of numbers "a", "b", "c" described above.

Similarly, the relative change in species frequency between two periods was counted as f2-f1 / max (f1, f2), where "f1" was the frequency of a species in the former period and "f2" the frequency of the species in the latter period.

Supplementary Table S1. The summary of "winners", i.e., species or OTU (operational taxonomic unit) that newly occurred or increased their mean abundance in 2015 (period 3) by ≥50% compared to 1997–2000 (period 1) in individual stream types along the river continuum: low altitude rivers (LowRiv), mid-altitude rivers (MidRiv), submontane rivers (SubRiv), mid-altitude brooks (MidBro), and submontane brooks (SubBro). The stream types in which a taxon was "winning" is indicated. Freq_tot –, Freq1/Freq3 – frequency in period 1 or 3 in all stream types, Med1/Med3 – median abundance in period 1 or 3 in all stream types, Max1/Max3 – maximum abundance in period 1 or 3 in all stream types. Only species with combined frequency ≥3 were included.

Species/OTU	Freq1	Med1	Max1	Freq3	Med3	Max3	Stream type
**Platyhelminthes**							
Dugesia gonocephala	41	4	161	51	39	550	LowRiv, MidRiv, SubBro
Girardia tigrina	1	4	4	6	5.5	19	LowRiv
Polycelis felina	10	23	100	12	54	304	MidRiv, SubRiv
**Mollusca**							
Ancylus fluviatilis	31	9	314	51	15	179	MidBro
Bithynia tentaculata	2	2	2	4	16.5	44	LowRiv
Bythinella austriaca	3	4	4	8	7	118	MidBro
Gyraulus albus	0	NA	0	6	4	24	LowRiv
Pisidium casertanum	17	2	48	49	6	424	LowRiv, MidRiv, SubRiv, MidBro, SubBro
Pisidium henslowanum	4	7	14	8	5	42	LowRiv
Pisidium nitidum	4	3.5	430	5	4	10	MidRiv
Pisidium personatum	9	2	4	41	5	39	LowRiv, MidRiv, SubRiv, MidBro, SubBro
Pisidium subtruncatum	10	11	54	32	14.5	172	LowRiv, MidRiv, SubRiv, MidBro, SubBro
Potamopyrgus antipodarum	0	NA	0	6	10.5	76	MidRiv
Radix balthica	1	3	3	8	10.5	110	MidRiv
**Clitellata**							
Aulodrilus japonicus/pluriseta	6	21	68	40	24	390	LowRiv, SubRiv, MidBro, SubBro

Bothrioneurum vejdovskyanum	1	3	3	8	28.5	272	LowRiv
Caspiobdella fadejewi	2	2	3	3	3	14	LowRiv
Cognettia glandulosa/sphagnetorum	2	3	4	34	17.5	160	LowRiv, MidRiv, SubRiv, MidBro, SubBro
Eiseniella tetraedra	28	4	22	47	5	44	MidRiv, SubRiv, MidBro, SubBro
Erpobdella vilnensis	6	3.5	6	8	3.5	9	SubBro
Fridericia/Henlea spp.	11	3	7	37	5	297	LowRiv, MidRiv, SubRiv, SubBro
Glossiphonia complanata/concolor	4	4.5	8	10	3	6	MidRiv
Haplotaxis gordioides	25	5	37	16	2	72	MidBro, SubBro
Limnodrilus claparedeanus	0	NA	0	9	17	61	LowRiv, MidRiv
Limnodrilus hoffmeisteri	1	94	94	35	28	432	LowRiv, MidRiv, SubRiv, MidBro
Lumbriculus variegatus	12	4	34	26	8.5	84	LowRiv, MidRiv, SubBro
Marionina sp.	0	NA	0	11	8	108	LowRiv, MidRiv
Nais alpina	15	6	848	28	4	176	LowRiv, MidRiv, MidBro
Nais bretscheri	4	36	56	7	4	328	LowRiv
Nais elinguis	13	26	676	18	20	584	MidRiv
Nais pardalis/stolci	0	NA	0	9	4	160	LowRiv, SubRiv
Ophidonais serpentina	1	6	6	13	4	344	LowRiv, MidRiv
Rhyacodrilus coccineus/pygmaeus_ Epirodrilus pygmaeus	1	2	2	13	15	621	LowRiv, MidRiv
Stylaria lacustris	2	13.5	24	8	22.5	344	LowRiv, MidRiv
Trichodrilus strandi	0	NA	0	6	50.5	110	MidBro
Tubifex ignotus	0	NA	0	15	8	99	MidRiv, SubRiv
Tubifex tubifex	4	21	52	16	10	161	LowRiv, MidRiv
**Crustacea**							
Asellus aquaticus	8	6	33	13	8	77	MidRiv
Gammarus fossarum	46	116	5927	48	554	4354	MidRiv

Niphargus sp.	0	NA	0	6	3	193	MidRiv
Proasellus coxalis	0	NA	0	5	3	20	LowRiv
**Ephemeroptera**							
Caenis luctuosa/macrura	5	12	34	14	7.5	461	LowRiv, MidRiv, MidBro
Centroptilum luteolum	10	40	352	50	11	369	LowRiv, MidRiv, SubRiv, MidBro, SubBro
Ecdyonurus helveticus Gr.	13	11	81	18	47	265	MidBro, SubBro
Ecdyonurus torrentis	28	8.5	97	37	18	636	SubBro
Ecdyonurus venosus	12	7	80	18	20	263	SubRiv, SubBro
Electrogena ujhelyii	7	4	21	12	21	191	MidBro
Ephemera danica	24	7.5	107	51	30	1093	LowRiv, SubRiv, MidBro, SubBro
Habrophlebia lauta	30	8	284	48	24.5	859	MidRiv, MidBro, SubBro
Heptagenia flava	2	7.5	12	4	13.5	59	LowRiv
Nigrobaetis muticus	38	18.5	501	61	55	530	LowRiv, MidRiv, SubRiv, SubBro
Paraleptophlebia submarginata	25	13	105	46	7	188	MidBro, SubBro
Potamanthus luteus	4	16.5	502	14	31	633	LowRiv, MidRiv
**Odonata**							
Calopteryx splendens	3	2	9	8	5	54	LowRiv
Calopteryx virgo	7	7	15	16	3.5	45	MidRiv
Cordulegaster boltonii	0	NA	0	7	2	10	MidRiv, MidBro
Gomphus vulgatissimus	3	2	5	10	3.5	16	LowRiv, MidRiv
Ischnura sp.	0	NA	0	3	2	9	LowRiv
Onychogomphus forcipatus	2	27	28	4	9	44	LowRiv
Platycnemis pennipes	0	NA	0	10	4.5	41	LowRiv, MidRiv
**Plecoptera**							
Amphinemura sulcicollis	20	12	94	20	26	278	MidBro
Brachyptera risi	14	22.5	239	32	8	697	LowRiv, MidRiv
Dinocras cephalotes	13	7	77	15	8	56	MidRiv, SubBro

Isoperla oxylepis/grammatica	46	22	156	52	33	184	LowRiv
Leuctra nigra	7	27	165	22	4	125	MidRiv, SubRiv, MidBro
Nemoura cinerea	9	13	69	15	20	131	MidBro
Nemoura marginata Gr.	35	10	140	43	20	194	SubRiv
Nemoura sciurus	1	1	1	6	5	48	MidBro
Perla abdominalis	4	5.5	17	11	20	106	SubRiv
Perla marginata	9	6	44	12	21	110	MidBro
Protonemura aestiva/auberti	6	25	50	13	16	175	MidBro
Protonemura meyeri	6	3	7	7	2	24	MidRiv
Protonemura praecox	1	2	2	4	5	11	SubBro
Siphonoperla taurica	2	11.5	21	4	4.5	74	MidRiv
**Heteroptera**							
Aphelocheirus aestivalis	9	6	13	13	83	418	LowRiv, MidRiv
Micronecta sp.	2	13	24	20	9	318	LowRiv, MidRiv, SubRiv
Velia caprai	0	NA	0	7	1	2	MidBro, SubBro
**Megaloptera**							
Sialis fuliginosa	9	1	4	31	3	42	MidRiv, SubRiv, MidBro, SubBro
**Coleoptera**							
Anacaena globulus	1	1	1	6	2	3	MidBro
Elodes/Odeles sp.	16	5	54	31	8	137	SubBro
Esolus angustatus/parallelepipedus	20	14	48	36	10	368	MidBro, SubBro
Gyrinus substriatus	0	NA	0	4	1	4	SubBro
Hydrocyphon deflexicollis	0	NA	0	10	15.5	87	MidRiv, MidBro, SubBro
Limnebius truncatellus	0	NA	0	11	2	6	MidBo, SubBro
Ochthebius melanescens	0	NA	0	4	6	24	MidRiv
Orectochilus villosus	16	8	64	33	32	167	MidRiv, SubRiv
Oreodytes sanmarkii	20	8.5	42	24	6.5	63	SubBro

Oulimnius tuberculatus	21	10	56	28	11	362	LowRiv, MidRiv
Platambus maculatus	10	4.5	13	48	5.5	97	LowRiv, MidRiv, SubRiv, MidBro, SubBro
Riolus subviolaceus	1	2	2	5	3	30	MidRiv
**Trichoptera**							
Agapetus ochripes	11	4	34	18	27	384	LowRiv, MidRiv, SubRiv, MidBro, SubBro
Allogamus auricollis	15	22	426	21	13	250	MidRiv
Anabolia furcata	3	1	11	10	5.5	61	LowRiv, SubRiv
Anomalopterygella chauviniana	13	31	205	17	31	296	MidRiv
Athripsodes albifrons	1	18	18	4	6	127	LowRiv
Athripsodes bilineatus	7	11	41	15	38	175	LowRiv, MidRiv, SubRiv, MidBro, SubBro
Athripsodes cinereus	7	5	30	13	10	280	LowRiv, MidRiv
Brachycentrus subnubilus	3	4	17	11	4	324	LowRiv, MidRiv
Ceraclea annulicornis	2	1.5	2	9	2	6	LowRiv, MidRiv
Cyrnus trimaculatus	2	2	2	7	4	9	MidRiv
Ecclisopteryx dalecarlica	20	20	492	24	15.5	139	MidBro
Ecclisopteryx madida	10	9	51	14	6	65	MidBro
Glossosoma conformis	21	4	101	25	8	63	MidRiv
Goera pilosa	1	2	2	7	2	6	LowRiv, MidRiv
Halesus digitatus/radiatus/tesselatus	34	8.5	88	47	15	146	LowRiv, MidRiv
Hydropsyche angustipennis	0	NA	0	4	2.5	16	LowRiv
Hydropsyche fulvipes	5	5	21	6	68	225	LowRiv
Hydropsyche instabilis	24	13	210	35	32	399	SubBro
Hydropsyche modesta	0	NA	0	4	18	67	LowRiv
Hydropsyche siltalai	29	41	902	38	41.5	1009	MidBro, SubBro
Hydroptila sp.	1	5	5	10	3.5	30	LowRiv, MidRiv
Chaetopteryx sp.	28	6	65	38	11	172	MidRiv
Cheumatopsyche lepida	7	46	1390	18	81.5	1462	MidRiv

Ithytrichia lamellaris	4	1.5	3	8	3.5	18	MidRiv
Lepidostoma basale	15	7	823	26	8.5	142	SubRiv
Lype reducta	2	2	2	18	3	8	MidRiv, SubRiv, SubBro
Micrasema longulum	11	6	104	14	4.5	50	MidRiv
Micrasema minimum	8	3	808	15	78	411	MidRiv
Micrasema setiferum	1	4	4	4	5	38	MidRiv
Mystacides azurea	11	3	27	31	9	59	LowRiv, MidRiv, SubRiv
Mystacides nigra	1	2	2	5	1	6	MidRiv
Odontocerum albicorne	25	3	53	42	9	132	MidRiv, MidBro, SubBro
Oecetis testacea	0	NA	0	8	4.5	12	MidRiv
Philopotamus montanus	9	6	143	18	14	124	MidRiv, MidBro
Plectrocnemia conspersa	19	2	24	23	9	279	MidBro
Polycentropus flavomaculatus	26	10	67	40	15	201	MidBro
Polycentropus irroratus	2	3.5	6	10	5	40	MidRiv, SubRiv
Potamophylax cingulatus/latipennis/luctuosus	32	6	188	48	14.5	151	MidRiv
Rhyacophila polonica/praemorsa	3	4	27	9	6	16	MidBro, SubBro
Sericostoma personatum/schneiderii	42	6.5	237	56	34.5	518	LowRiv, MidRiv, SubRiv, MidBro, SubBro
Silo nigricornis/piceus	14	6	31	18	5	141	MidRiv
Silo pallipes	18	6.5	47	29	4	67	MidBro
Synagapetus iridipennis	0	NA	0	3	8	10	MidBro
Tinodes sp.	16	2.5	17	26	6	103	MidRiv, SubRiv, MidBro, SubBro
**Diptera**							
Antocha vitripennis	16	3	12	24	4	76	MidRiv
Atrichops crassipes	1	20	20	5	7	72	LowRiv
Berdeniella sp.	1	2	2	24	9	123	MidRiv, SubRiv, MidBro, SubBro
Ceratopogoninae Gen. sp.	30	4.5	56	60	9	105	SubRiv, MidBro, SubBro

Dixa sp.	3	4	16	28	3	27	MidRiv, MidBro, SubBro
Ellipteroides alboscutellatus/lateralis	0	NA	0	9	4	49	MidBro, SubBro
Eloeophila sp.	29	2	16	43	10	61	MidBro, SubBro
Hemerodromia sp.	3	2	4	28	3.5	40	LowRiv, MidRiv, SubRiv, MidBro
Hexatoma sp.	30	9.5	202	42	22.5	121	MidBro, SubBro
Chelifera sp.	2	3	4	39	6	67	LowRiv, MidRiv, SubRiv, MidBro, SubBro
Chrysops caecutiens	2	3.5	6	19	4	58	MidRiv, SubRiv, MidBro, SubBro
Ibisia marginata	26	6.5	141	38	11.5	352	SubRiv, SubBro
Jungiella sp.	0	NA	0	4	2	4	MidBro
Limnophora sp.	1	1	1	11	3	13	LowRiv, MidRiv, SubRiv
Oxycera pygmaea	1	1	1	4	2.5	7	MidBro
Pedicia sp.	9	2	153	19	3	14	MidBro, SubBro
Pericoma sp.	8	3.5	31	35	4	150	LowRiv, MidRiv, SubRiv, MidBro, SubBro
Pneumia sp.	1	2	2	14	2	52	MidBro, SubBro
Pseudolimnophila sp.	0	NA	0	7	2	34	MidRiv
Ptychoptera sp.	0	NA	0	11	4	42	MidBro, SubBro
Tabanus sp.	1	2	2	6	2	3	SubBro
Tachytrechus sp.	0	NA	0	5	2	23	LowRiv
Tipula sp.	29	3	17	53	3	36	SubRiv, SubBro
Wiedemannia sp.	24	5	216	32	6	34	MidRiv

Supplementary Table S2. The summary of "losers", i.e., species or OTU (operational taxonomic unit) that disappeared or decreased their mean abundance in 2015 (period 3) by ≥50% compared to 1997–2000 (period 1) in individual stream types along the river continuum: low altitude rivers (LowRiv), mid-altitude rivers (MidRiv), submontane rivers (SubRiv), mid-altitude brooks (MidBro), and submontane brooks (SubBro). The stream types in which a taxon was "losing" is indicated. Freq1/Freq3 – frequency in period 1 or 3 in all stream types, Med1/Med3 – median abundance in period 1 or 3 in all stream types, Max1/Max3 – maximum abundance in period 1 or 3 in all stream types. Only species with combined frequency ≥3 were included. Please note that for some "losers" the values in period 3 might increase compared to period 1 because they did not "lose" in the whole river continuum; the same taxon may become a "loser" in one stream type and a "winner" in another.

Species/OTU	Freq1	Med1	Max1	Freq3	Med3	Max3	Stream type
**Clitellata**							
Nais alpina	15	6	848	28	4	176	SubBro
Nais communis	7	12	184	17	2	60	SubBro
Potamothrix hammoniensis	10	9.5	68	9	10	64	MidRiv
Haplotaxis gordioides	25	5	37	16	2	72	LowRiv, MidRiv, SubRiv
**Ephemeroptera**							
Baetis alpinus	27	27	2443	23	20	742	MidRiv
Baetis vernus	14	25.5	330	14	4	244	LowRiv
Cloeon dipterum s. lat.	5	4	8	2	4.5	5	LowRiv
Ecdyonurus starmachi	8	35.5	247	5	20	46	MidRiv
Ecdyonurus submontanus	24	20	528	10	16.5	285	LowRiv, MidRiv, SubRiv
Epeorus assimilis	44	15.5	309	40	53.5	573	LowRiv
Rhithrogena carpatoalpina/iridina/picteti	53	85	1731	43	150	788	LowRiv
Rhithrogena semicolorata	47	64	1647	38	52.5	751	MidBro, SubBro
Habrophlebia fusca	8	4	84	2	80.5	159	SubRiv, SubRiv
Ephemerella ignita	28	5.5	76	28	2.5	13	SubRiv
**Plecoptera**							

Isoperla difformis	4	4.5	6	2	10	18	SubRiv
Isoperla sudetica	9	2	20	3	3	14	SubBro
Chloroperla tripunctata	5	18	93	1	16	16	SubRiv
Taeniopteryx hubaulti	7	8	26	3	4	22	SubRiv
Brachyptera seticornis	27	26	156	23	8	108	SubBro
Leuctra autumnalis	4	7.5	40	0	NA	0	MidBro
Leuctra prima Gr.	5	6	56	2	21	30	SubBro
**Coleoptera**							
Oulimnius tuberculatus	21	10	56	28	11	362	MidBro, SubBro
**Trichoptera**							
Rhyacophila tristis	21	6	20	19	13	67	SubRiv
Glossosoma intermedium	4	1.5	4	2	2.5	3	SubRiv
Hydropsyche instabilis	24	13	210	35	32	399	LowRiv
Hydropsyche saxonica	28	9	166	22	3	61	MidRiv
Drusus discolor	5	6	64	1	5	5	SubBro
Lepidostoma basale	15	7	823	26	8.5	142	LowRiv
Beraeodes minutus	4	1	4	2	6	10	MidRiv
**Diptera**							
Eloeophila sp.	29	2	16	43	10	61	LowRiv
Ormosia sp.	4	3	4	0	NA	0	SubBro
Rhypholophus haemorrhoidalis	6	2	14	5	2	2	MidBro
Liponeura cinerascens/cordata	5	3	11	3	3	34	MidBro
Psychoda sp.	7	8	44	3	2	5	SubRiv
Tonnoiriella sp.	7	10	19	1	2	2	SubBro
Wiedemannia sp.	24	5	216	32	6	34	MidBro
Clinocera sp.	9	4	14	0	NA	0	LowRiv

Supplementary Table S3. Changes in selected environmental variables between 1997–2000 (period 1) and 2015 (period 3) in five stream types along a river continuum: low altitude rivers (LowRiv), mid-altitude rivers (MidRiv), submontane rivers (SubRiv), mid-altitude brooks (MidBro), and submontane brooks (SubBro), tested by non-parametric Wilcoxon U pair test. T_Jan - mean air temperatures in January, T_Jul - mean air temperatures in July, Prec - annual precipitation sums, Phi - roughness of bed substrate, NO_3 - concentration of nitrates, Riff - share of riffles, Fore - forested area, Un_surf - unfavourable surfaces, TP - concentration of total phosphorus, BOD_5 - biological oxygen demand, NH_4 - concentration of ammonium cations.

**Enviromental variable**	**All**		**LowRiv**		**MidRiv**		**SubRiv**		**MidBro**		**SubBro**	
	**change**	**p**	**change**	**p**	**change**	**p**	**change**	**p**	**change**	**p**	**change**	**p**
**T_Jan**	increase	0.000	increase	0.008	increase	0.000	increase	0.005	increase	0.001	increase	0.000
**T_Jul**	increase	0.000	increase	0.008	increase	0.000	increase	0.005	increase	0.001	increase	0.000
**Prec**	decrease	0.000	no	0.953	decrease	0.013	no	0.959	decrease	0.022	decrease	0.006
**Phi**	no	0.084	increase	0.028	no	0.496	no	0.575	increase	0.022	no	0.535
**NO_3**	decrease	0.007	decrease	0.021	decrease	0.011	decrease	0.022	no	0.177	no	0.196
**Riff**	increase	0.004	no	1.000	increase	0.010	increase	0.028	no	0.638	no	0.307
**Fore**	increase	0.000	increase	0.028	no	0.061	no	0.241	no	0.051	increase	0.001
**Un_surf**	decrease	0.000	decrease	0.012	decrease	0.002	no	0.093	decrease	0.046	no	0.345
**TP**	no	0.563	decrease	0.008	no	0.605	no	0.878	no	0.177	no	0.177
**BOD_5**	no	0.099	no	0.859	no	0.605	no	0.169	increase	0.011	no	0.211
**NH_4**	no	0.283	no	0.086	no	0.569	no	0.093	no	0.975	decrease	0.036

Supplementary Figure S1. The distributions of relative change in abundance and frequency of macroinvertebrate species between 1997–2000 (period 1) and 2015 (period 3) in five stream types along a river continuum: low altitude rivers (LowRiv), mid-altitude rivers (MidRiv), submontane rivers (SubRiv), mid-altitude brooks (MidBro), and submontane brooks (SubBro). For calculations of the relative change see Supplements to Methods.

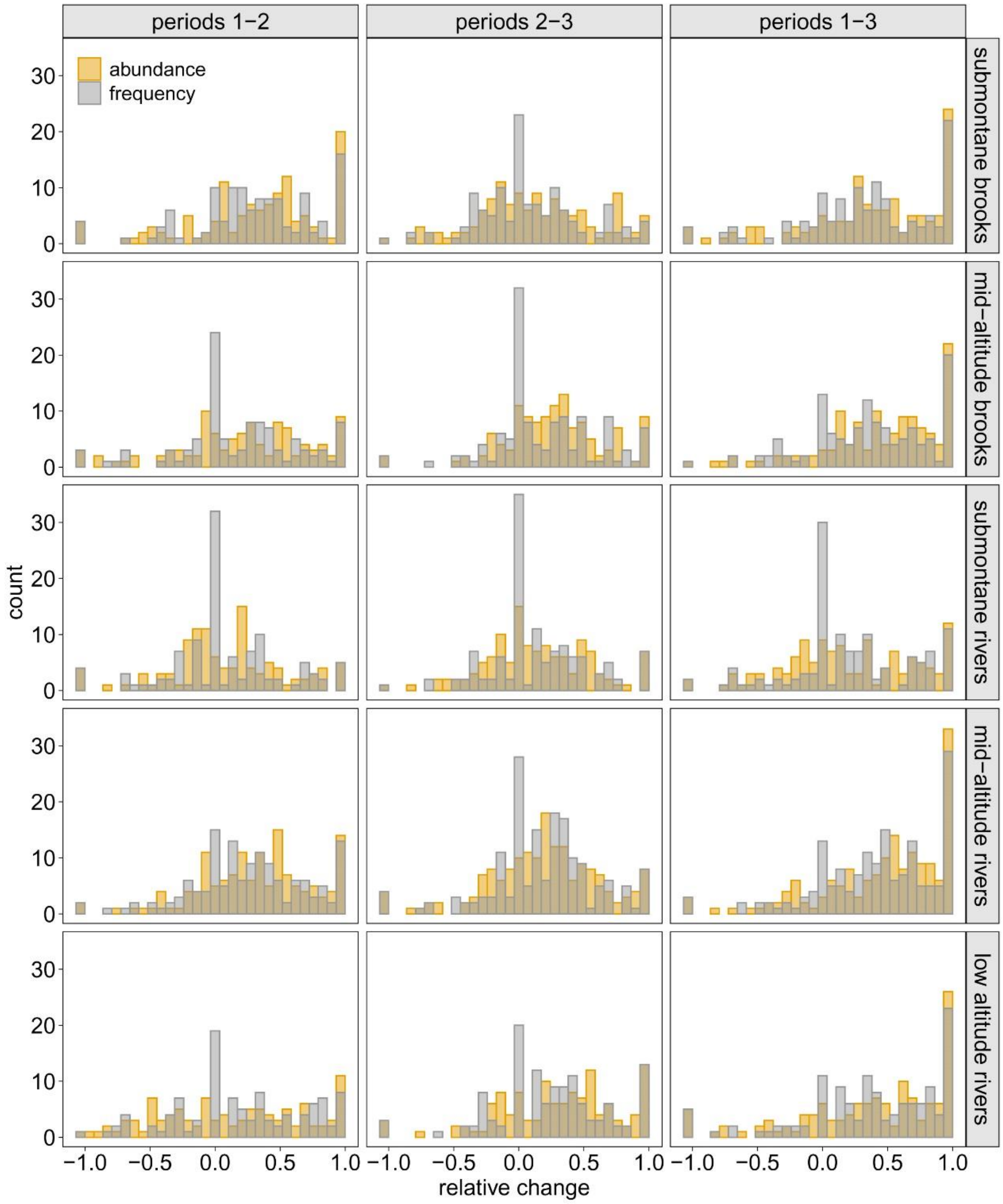

Supplementary Figure S2. Changes in selected significant environmental variables at individual sites among three sampling periods (1997–2000, 2007–2008, and 2015) in five stream types along a river continuum: low altitude rivers (LowRiv), mid-altitude rivers (MidRiv), submontane rivers (SubRiv), mid-altitude brooks (MidBro), and submontane brooks (SubBro). T_Jan - mean air temperatures in January, T_Jul - mean air temperatures in July, Prec - annual precipitation, Phi - roughness of bed substrate, NO_3 - concentration of nitrates, Riff - share of riffles, Fore - forested area, Un_surf - unfavourable surfaces.

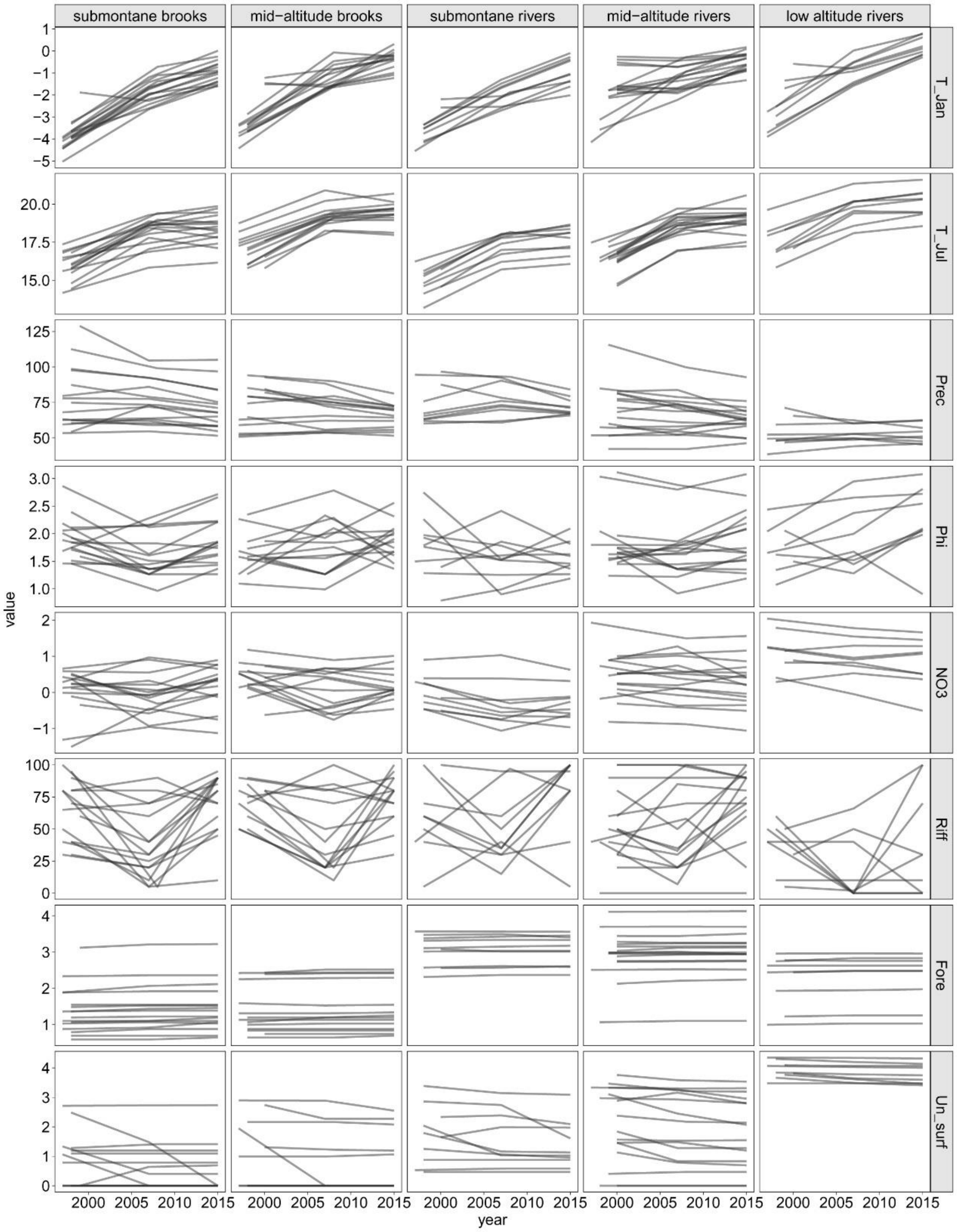

Supplement: Supplementary file 1 — Supplementary Information. [file 41598_2023_32806_MOESM1_ESM.pdf]
